# Wheat phyllosphere yeasts degrade propiconazole

**DOI:** 10.1186/s12866-020-01885-6

**Published:** 2020-08-05

**Authors:** Katarzyna Kucharska, Urszula Wachowska, Sylwester Czaplicki

**Affiliations:** 1 Department of Entomology, Phytopathology and Molecular Diagnostics, Faculty of Environmental Management and Agriculture, University of Warmia and Mazury in Olsztyn, Olsztyn, Poland; 2 Department of Food Plant Chemistry and Processing, Faculty of Food Sciences, University of Warmia and Mazury in Olsztyn, pl. Cieszyński 1, 10-726 Olsztyn, Poland

**Keywords:** Wheat leaves, Fungicide, *Aureobasidium*, *Rhodotorula*

## Abstract

**Background:**

Yeasts, which are ubiquitous in agroecosystems, are known to degrade various xenobiotics. The aim of this study was to analyze the effect of fungicides on the abundance of natural yeast communities colonizing winter wheat leaves, to evaluate the sensitivity of yeast isolates to fungicides in vivo, and to select yeasts that degrade propiconazole.

**Results:**

Fungicides applied during the growing season generally did not affect the counts of endophytic yeasts colonizing wheat leaves. Propiconazole and a commercial mixture of flusilazole and carbendazim decreased the counts of epiphytic yeasts, but the size of the yeast community was restored after 10 days. Epoxiconazole and a commercial mixture of fluoxastrobin and prothioconazole clearly stimulated epiphyte growth. The predominant species isolated from leaves were *Aureobasidium pullulans* and *Rhodotorula glutinis.* In the disk diffusion test, 14 out of 75 yeast isolates were not sensitive to any of the tested fungicides. After 48 h of incubation in an aqueous solution of propiconazole, the *Rhodotorula glutinis* Rg 55 isolate degraded the fungicide in 75%. Isolates *Rh. glutinis* Rg 92 and Rg 55 minimized the phytotoxic effects of propiconazole under greenhouse conditions. The first isolate contributed to an increase in the dry matter content of wheat seedlings, whereas the other reduced the severity of chlorosis.

**Conclusion:**

Not sensitivity of many yeast colonizing wheat leaves on the fungicides and the potential of isolate *Rhodotorula glutinis* Rg 55 to degrade of propiconazole was established. Yeast may partially eliminate the ecologically negative effect of fungicides.

## Background

Yeasts, which are ubiquitous in agroecosystems, are also widely used in industry, biotechnological research and food production [1–3]. The phyllosphere, which is defined as the above-ground part of a plant as a habit for microorganisms, is relatively deficient in nutrients and is not readily colonized by bacteria and fungi [4]. Yeast communities colonizing wheat leaves were initially described as “pink yeasts” of the genera *Sporobolomyces* and *Rhodotorula* and “white yeasts” of the genus *Cryptococcus* [5–7]. The predominance of yeast genera *Sporobolomyces* and *Cryptococcus* on wheat leaves was confirmed by next generation sequencing methods in contemporary research [8, 9]. These innovative experiments also support detailed descriptions of yeast communities. Previously undetected yeast species, including *Dioszegia crocea, D. aurantiaca, D. fristingensis, D. hungarica* [9] and *Vishniacozyma victoriae* [8], were identified on wheat leaves. Yeast communities respond selectively to abiotic (temperature, precipitation, pesticides) and biotic factors [8, 9].

Foliar fungicides are routinely applied in agriculture. They eliminate pathogens, but they can also exert negative effects on non-target fungi. This phenomenon has been monitored for several decades under field conditions. The fungicides applied in the 1980s, such as dithiocarbamates, captafol, benzimidazoles and tridemorph, reduce saprophytic microflora over a period of 2–3 weeks after the treatment [5]. In a study by Fokkema et al. [6], carbendazim decreased yeast counts in the short term, but these communities subsequently re-emerged by developing resistant strains. Azoxystrobin led to a significant increase in yeast abundance on wheat leaves [10]. In a detailed analysis conducted by Karrson et al. [9], fungicides acted selectively on various yeast species. For example, the proportion of *Sporobolomyces roseus* increased, whereas the proportion of *D. hungarica* decreased after the treatment. Fungicides’ effects on non-target saprotrophic fungi have to be explored because these microorganisms have a high potential for inhibiting the growth of plant pathogens [11]. However, these effects are difficult to monitor under field conditions because saprotrophs are influenced by numerous factors, including weather, growth stage and the health status of the protected plants [7]. In vitro studies provide valuable information about the sensitivity of various yeast species to fungicides. In a study conducted by Southwell et al. [12] under strictly controlled conditions, *Rhodotorula glutinis* was significantly more sensitive to mancozeb than *Aureobasidium pullulan* and *Cryptococcus albidus*. Yeast strains *Rh. glutinis*, *Cryptococcus laurentii* and *A. pullulans* used by Lima et al. [13] as antagonists of apple pathogens were sensitive to triazoles (penconazole and tebuconazole) and resistant to procymidone, vinclozolin, copper and oxychloride. In a study by Wachowska [14], a strobilurin fungicide strongly inhibited the development of *Sporobolomyces roseus, Candida tropicalis* and *Pichia anomala.*

According to Chandran and Das [15], yeast species *C. tropicalis*, *Cryptococcus laurentii*, *Trichosporon asahii*, *Rhodotorula mucilaginosa* and *Candida rugosa* with active cytochrome P450 enzymes participate in the degradation of diesel oil in soil. Selected strains of *Candidia methanosorbosa* degraded anilines, the putative degradation products of azo dyes [16]. Some species of yeasts and bacteria can transform organic aromatic pollutants, including polycyclic aromatic hydrocarbons, biphenyls, dibenzofurans and pesticides, through cometabolism [17]. *Streptomyces albogiseolus* and *Brevibacillus borstelensis* minimized environmental contamination with carbendazim [18]. Yeasts also play an important role in eliminating toxic heavy metals [19]. Bempelou et al. [20] demonstrated that *Rh. glutinis* and *Rh. rubra* degraded diazinon. In a study by Evy et al. [21], anthracene was broken down by *Pichia kudriavzevii* and *C. laurentii*. Dordević and Durović-Pejčev [22] reported on the ability of *Saccharomyces cerevisiae* to degrade pirimiphos-methyl. However, there are no published data on yeasts ability to degrade propiconazole.

Propiconazole (1-[[2-(2,4-dichlorophenyl)-4-propyl-1,3-dioxolan-2-yl]methyl]-1H-1,2,4-triazole) belongs to the triazole group of fungicides that block the biosynthesis of ergosterol in fungal cell membranes by inhibiting lanosterol 14α-demethylase, a cytochrome P450 enzyme. This fungicide is widely used in agriculture to protect cereals against pathogens [23], and its toxicity for mammals is generally regarded as low [24]. However, higher doses of propiconazole can decrease the synthesis of CYP51 protein (lanosterol 14α-demethylase) in both fungi and animals, which inhibits the synthesis of ergosterol and cholesterol [25, 26]. Propiconazole exerts carcinogenic effects in mice [27], and it can affect the hormonal status of animals and damage DNA [28, 29]. Propiconazole has a long half-life of 214–315 days in soil [30, 31]. It has a strong affinity for soil (soil adsorption coefficient Koc of 1800), therefore its concentration in agricultural runoffs is generally low in the year of treatment [32]. However, Riise et al. [30] found that the content of propiconazole residues in soil runoffs increases in the spring following the year of fungicide treatment. Propiconazole applied in various doses under laboratory conditions exerted phytotoxic effects on wheat seedlings by inhibiting root growth and reducing the respiration rate [31]. Fungicides containing propiconazole can also be toxic for plants in the field [32].

Various methods are used to remove contaminants from arable land, including contaminant saturation, recycling, pyrolysis and incineration [33, 34]. However, physicochemical remedy methods are expensive and not highly effective [35]. For this reason, pollutant-degrading microorganisms show considerable promise in environmental protection strategies [36–38]. Crop protection products can be removed from the environment by biotransformation, biomineralization, bioaccumulation, biodegradation and cometabolism [36, 37, 39]. Fungi and bacteria secret extracellular enzymes under exposure to environmental stressors. Enzymes such as transferase, isomerase, hydrolase and ligase participate in the breakdown of crop protection agents. Degradation processes involve mainly bacteria, but fungi including yeasts also play an important role by decomposing contaminants that cannot be broken down by bacteria [36, 40]. Cytochrome P450 enzymes enable fungi to adapt to adverse conditions and degrade a wide range of environmental toxins [41].

The aim of this study was to analyze the effect of fungicides on the abundance of natural fungal communities colonizing winter wheat leaves in a field experiment, to evaluate the sensitivity of yeast isolates to fungicides in vitro, and to select yeasts with a high potential for degrading propiconazole to protect wheat seedlings against the phytotoxic effects of this compound.

## Results

### Yeast counts on wheat leaves protected with fungicides

**Fig. 1:**
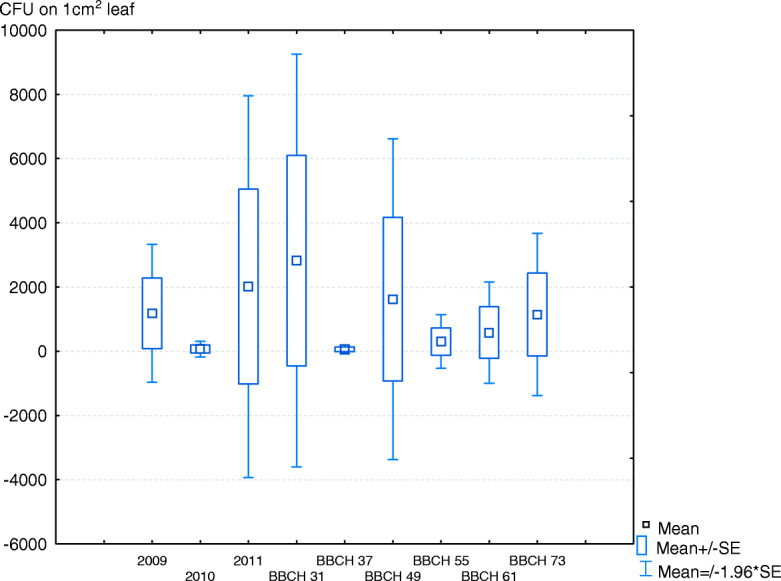
Mean counts of yeasts isolated from leaves during three years of the study and during six wheat growth stages (BBCH, refer to Table 2). CFU – colony-forming unit, SE – standard error. – Mean, − Mean +/−SE, − Mean +/− 1.96^·•^ SE

Yeasts were isolated from 90.8% of leaf samples. All experimental factors (year, growth stage, protective treatment) significantly influenced yeast counts (Table 1). In 2009, 2010 and 2011, the average yeast counts on leaves were determined at 1073.1, 61.3 and 2091.7 CFU, respectively (Fig. 1). The average yeast counts were determined at 3049.12 CFU in stage BBCH 31, but they reached only 61.0 CFU in stage BBCH 37 (Fig. 1). Precipitation exerted a minor negative effect on yeast abundance on leaves because the correlation coefficient was determined at − 0.33.

**Table 1:** Three-way ANOVA of yeast counts

Factor	df	F
Year (Y)	2	328.67**
Date (D)	5	95.51**
Treatment (T)	2	3.62*
Y x D	10	89.23**
Y x T	4	8.49**
D x T	10	10.0**
Y x D x T	20	2.48**

** - differ significantly at *p* < 0.001, * - differ significantly at *p* < 0.005

**Fig. 2:**
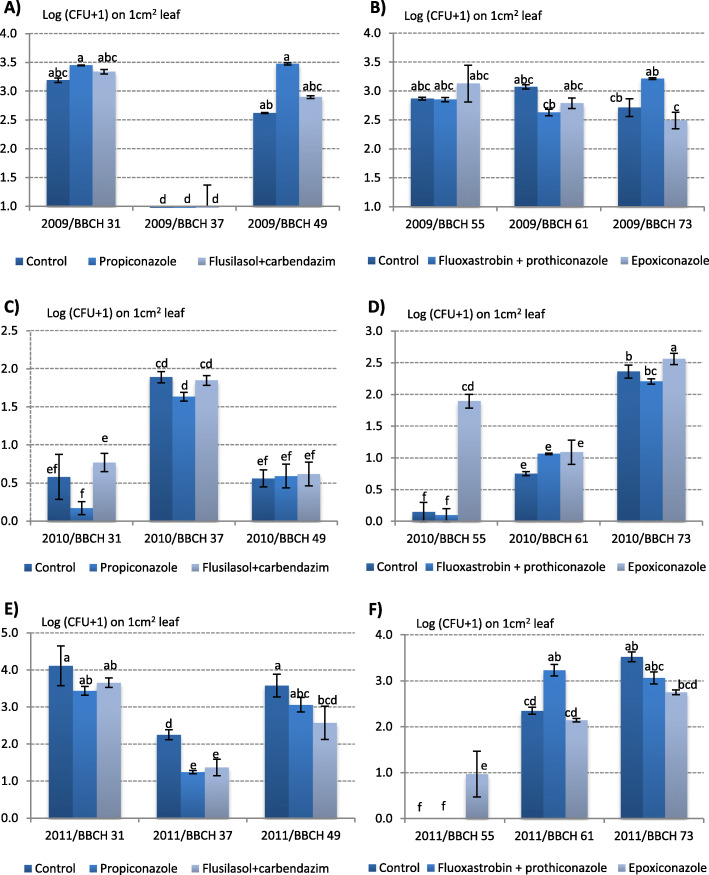
Size of yeast communities isolated from wheat leaves after fungicide treatments applied in stages BBCH 31 (A, C, D) and BBCH 55 (B, D, F) during three years of the study (BBCH stages are described Table 2). Values that did not differ significantly in the SNK test (*p* < 0.001) are marked with the same letters

Fungicides strongly reduced the total size of yeast communities only in 2011 (Fig. 2). Propiconazole decreased yeast counts in stage BBC 37 relative to control, and yeasts were effectively eliminated by the commercial fungicide containing flusilazole and carbendazim in stages BBCH 37 and 49 (Fig. 2e). In stage BBCH 73, yeasts were far less abundant on leaves treated with epoxiconazole than on unprotected leaves (Fig. 2f). In very few cases, fungicides contributed to a significant increase in yeast counts (Fig. 2d, f).

### Yeast identification

Six yeast species and genera were isolated from wheat leaves: *A. pullulans* (De Bary) G. Arnaud, *Rh. glutinis* (Fresenius) F.C. Harrison, *Cryptococcus* sp., *Pseudozyma* sp., *Debaryomyces hansenii* (Zopf) Lodder & Kreger-van Rij (anamorph: *Candida famata* (Harrison) S. A Meyer & Yarrow var. *famata*) and *C. albicans* (Robin) Berkhout (Table 2). They were identified based on the sequences of ITS1, 5.8S and ITS2 rDNA fragments which were characterized by more than 99% homology with the corresponding regions in most yeast isolates. *Cryptococcus* sp. isolates were not similar to any known species of this genus in the available databases. *Aureobasidium pullulans* belongs to the division Ascomycota, subdivision Pezizomycotina*. Debaryomyces hansenii* and *C. albicans* belonged to the division Ascomycota, subdivision Saccharomycotina. The division Basidiomycota was represented by *Rh. glutinis, Rh. pinicola, Pseudozyma* sp. and *Cryptococcus* sp. The ITS1, 5.8S and ITS2 rDNA sequences of some isolates were deposited in GenBank under the following accession numbers: *A. pullulans* - KX444670, *D. hansenii* - KX444669, *C. albicans* - KX444661, *Rh. glutinis* - KX424655.

**Table 2:** Yeast strains isolated from wheat leaves and used in the disk diffusion test (A) and the survival test in a liquid medium with propiconazole (B)

Isolate	Origin			Year of isolation	Sensitivity to fungicides in the disk diffusion test ^A^	Survival in a liquid medium ^B^
	En/Ep ^∂^	Treatment ^Ψ^	Wheat growth stage			
*Aureobasidium pullulans* 14	En	Control	BBCH 49	2009	S	–
*Aureobasidium pullulans* 15	En	Control	BBCH 49	2009	HS	–
*Aureobasidium pullulans* 16	En	Control	BBCH 49	2009	HS	–
*Aureobasidium pullulans* 17	En	Control	BBCH 49	2009	HS	–
*Rhodotorula glutinis* 18	En	Control	BBCH 49	2009	S	–
*Aureobasidium pullulans* 19	En	Control	BBCH 49	2009	S	–
*Rhodotorula glutinis* 39	En	Fung 1	BBCH 49	2009	S	–
*Aureobasidium pullulans* 40	En	Fung 1	BBCH 49	2009	HS	–
*Aureobasidium pullulans* 41	En	Fung 1	BBCH 49	2009	HS	–
*Aureobasidium pullulans* 42	En	Fung 1	BBCH 49	2009	HS	–
*Aureobasidium pullulans* 43	En	Fung 1	BBCH49	2009	S	–
*Aureobasidium pullulans* 44	En	Fung 1	BBCH49	2009	S	–
*Aureobasidium pullulans* 45	En	Fung 1	BBCH 49	2009	HS	–
*Aureobasidium pullulans* 46	En	Fung 1	BBCH 49	2009	S	–
*Aureobasidium pullulans* 47	En	Fung 1	BBCH 49	2009	HS	–
*Rhodotorula glutinis* 48	En	Control	BBCH 55	2009	S	–
*Aureobasidium pullulans* 49	En	Control	BBCH 55	2009	S	–
*Rhodotorula glutinis* 50	En	Control	BBCH 55	2009	S	–
*Rhodotorula glutinis* 51	En	Control	BBCH 55	2009	NS	–
*Rhodotorula glutinis* 52	En	Control	BBCH 55	2009	NS	100
*Aureobasidium pullulans* 53	En	Control	BBCH 55	2009	S	–
*Cryprococcus* sp. 54	En	Control	BBCH 55	2009	S	–
*Rhodotorula glutinis* Rg 55	En	Control	BBCH 55	2009	NS	100
*Rhodotorula glutinis* 56	En	Control	BBCH 55	2009	NS	–
*Rhodotorulla glutinis* 57	En	Control	BBCH 55	2009	NS	100
*Aureobasidium pullulans* 58	En	Control	BBCH 55	2009	NS	–
*Aureobasidium pullulans* 59	En	Control	BBCH 55	2009	S	200
*Aureobasidium pullulans* 60	En	Control	BBCH 55	2009	S	–
*Aureobasidium pullulans* 61	En	Control	BBCH 55	2009	HS	–
*Aureobasidium pullulans* 62	En	Control	BBCH 55	2009	HS	–
*Aureobasidium pullulans* 63	En	Control	BBCH 55	2009	HS	–
*Aureobasidium pullulans* 81	En	Fung 3	BBCH 55	2009	S	100
*Aureobasidium pullulans* 82	En	Fung 3	BBCH 55	2009	S	–
*Aureobasidium pullulans* 83	En	Fung 3	BBCH 55	2009	NS	–
*Aureobasidium pullulans* 84	En	Fung 4	BBCH 55	2009	S	–
*Aureobasidium pullulans* 85	En	Fung 4	BBCH 55	2009	S	–
*Aureobasidium pullulans* 86	En	Fung 4	BBCH 55	2009	S	–
*Rhodotorula glutinis* 87	En	Fung 4	BBCH 55	2009	HS	–
*Rhodotorula glutinis* 88	En	Fung 4	BBCH 55	2009	S	–
*Aureobasidium pullulans* 89	En	Fung 4	BBCH 55	2009	HS	–
*Aureobasidium pullulans* 90	En	Fung 4	BBCH 55	2009	HS	–
*Aureobasidium pullulans* 91	En	Fung 4	BBCH 55	2009	NS	–
*Rhodotorula glutinis* Rg 92	En	Fung 4	BBCH 55	2009	NS	100
*Rhodotorula glutinis* 102	Ep	Fung 4	BBCH 61	2009	S	–
*Rhodotorula glutinis* 103	Ep	Fung 4	BBCH 61	2009	NS	100
*Aureobasidium pullulans* 104	Ep	Fung 4	BBCH 61	2009	HS	–
*Aureobasidium pullulans* 105	Ep	Fung 4	BBCH 61	2009	S	–
*Rhodotorula glutinis* 111	Ep	Control	BBCH 73	2009	S	–
*Aureobasidium pullulans* 113	Ep	Fung 4	BBCH 73	2009	NS	–
*Aureobasidium pullulans* 132	En	Fung 1	BBCH 37	2010	S	–
*Aureobasidium pullulans* 135	Ep	Fung 1	BBCH 37	2010	S	–
*Rhodotorula glutinis*136	En	Fung 2	BBCH 37	2010	NS	100
*Aureobasidium pullulans* 137	Ep	Control	BBCH 49	2010	NS	–
*Cryptococcus* sp.138	Ep	Control	BBCH 49	2010	S	–
*Debaryomyces hansenii* 154	Ep	Fung 3	BBCH 55	2010	S	–
*Candida albicans*155	Ep	Fung 3	BBCH 55	2010	S	–
*Cryptococcus* sp. 163	En	Fung 3	BBCH 61	2010	NS	100
*Debaryomyces hansenii* 164	En	Fung 4	BBCH 61	2010	S	–
*Aureobasidium pullulans* 166	En	Fung 4	BBCH 61	2010	S	–
*Aureobasidium pullulans* 168	Ep	Fung 4	BBCH 61	2010	S	–
*Rhodotorula glutinis* 176	En	Fung 2	BBCH 31	2011	NS	100
*Rhodotorula glutinis* 177	En	Fung 2	BBCH 31	2011	NS	100
*Aureobasidium pullulans* 185	En	Fung 1	BBCH 37	2011	S	–
*Pseudozyma* sp.186	Ep	Fung 2	BBCH 37	2011	HS	–
*Aureobasidium pullulans* 187	En	Control	BBCH 49	2011	S	–
*Aureobasidium pullulans* 190	En	Fung 1	BBCH 49	2011	S	–
*Aureobasidium pullulans* 191	En	Fung 1	BBCH 49	2011	NS	–
*Aureobasidium pullulans* 203	En	Control	BBCH 61	2011	NS	100
*Aureobasidium pullulans* 210	Ep	Fung 4	BBCH 73	2011	S	–
*Aureobasidium pullulans* 211	En	Fung 4	BBCH 73	2011	HS	–
*Cryptococcus* sp*.* 212	Ep	Fung 3	BBCH 73	2011	S	–
*Cryptococcus* sp. 48	En	Control	BBCH 73	2010	NS	100
*Cryptococcus* sp. C 123	En	Control	BBCH 73	2011	NS	100
*Aureobasidium pullulans* 83	Ep	Control	BBCH 73	2010	NS	100
*Aureobasidium pullulans* 137	Ep	Control	BBCH 73	2011	NS	100

^∂^ En - endophyte, Ep – epiphyte, ^Y^ refer to Table 5, ^A^ NS – non-sensitive, no inhibition zone around discs saturated with all tested fungicides; S – sensitive, inhibition zone of 1–250 mm^2^; HS – highly sensitive, inhibition zone > 250 mm^2^

^B^ 100 – isolates growing in a liquid medium containing 100 μl dm^−3^ of propiconazole, 200 – isolates growing in a liquid medium containing 200 μl dm^−3^ of propiconazole, “-” – not analyzed

BBCH 31 - First node at least 1 cm above tillering node; BBCH 37 - flag leaf just visible, still rolled; BBCH 49 - first awns visible (in awned forms only); BBCH 55 - middle of heading: half of inflorescence emerged; BBCH 61 - beginning of flowering: first anthers visible; BBCH 73 - early milk; BBCH 93 - grains loosening in day-time

### Sensitivity of yeast isolates in the disk diffusion test

**Fig. 3:**
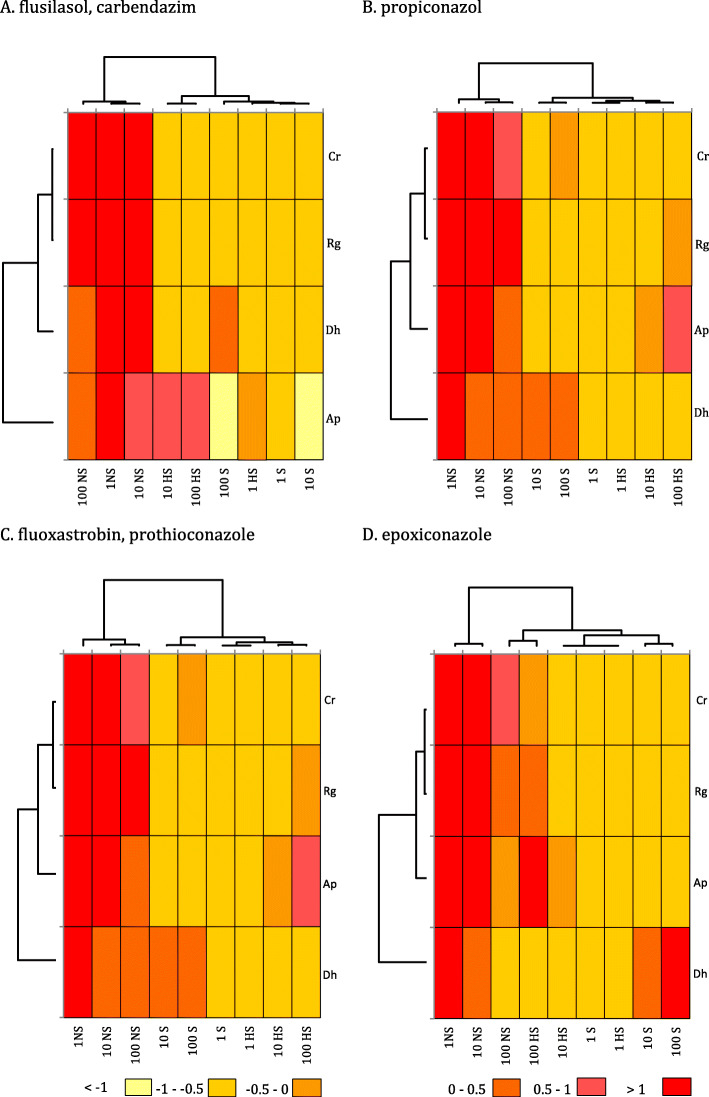
Percentage of yeast isolates that were non-sensitive (NS), sensitive (S) and highly sensitive (HS) to fungicide doses of 1, 10 and 100 μm dm^− 3^. Ap – *Aureobasidium pullulans*, Rg - *Rhodotorula glutinis*, Dh - *Debaryomyces hansenii*, Cr - *Cryptococcus* sp. Scale: > 1 – all tested isolates were sensitive to the fungicide; 0.5–1 – 80-99% of the isolates were sensitive to the applied fungicide concentration; 0–0.5 – 60-79% of the isolates were sensitive to the applied fungicide concentration; − 0.5 - 0 – 40-59% of the isolates were sensitive to the applied fungicide concentration; − 1 - 0.5 – 1-39% of the isolates were sensitive to the applied fungicide concentration; < − 1 – none of the tested isolates were sensitive to the fungicide

The predominant yeast species in the group of 75 isolates were *A. pullulans* and *Rh. glutinis* (Table 2). Fourteen isolates did not respond to any of the tested fungicides at any concentration. Inhibition zones were clearly smaller around discs saturated with a commercial mixture of fluoxastrobin and propiconazole at 100 μl dm^− 3^ than around discs saturated with propiconazole and epoxiconazole (Table 3). The percentage of *A. pullulans* isolates not sensitive to the lowest fungicide dose ranged from 63.6% (commercial mixture of flusilazole and carbendazim) to 97.7% (commercial mixture of fluoxastrobin and propiconazole) (Fig. 3). Only 18.2% of *A. pullulans* were insensitive to an epoxiconazole dose of 100 μm dm^− 3^. Isolates *Rh. glutinis*, *D. hansenii* and *Cryptococcus* sp. were even less sensitive to fungicides. The percentage of isolates that did not respond to the lowest concentrations of the tested fungicides ranged from 94.7% (*Rh. glutinis*) to 100% (*D. hansenii*, *Cryptococcus* sp.) (Fig. 3).

**Table 3:** Average sensitivity of yeast isolates to fungicides in the disk diffusion test

Dose (μl per 1 L)	Flusilazole, carbendazim	Propiconazole	Fluoxastrobin, prothioconazole	Epoxiconazole
	Area of inhibition zone (mm^2^)			
1	67.75 ^*de*^	12.68 ^*e*^	1.17 ^*e*^	28.78 ^*e*^
10	170.14 ^*cd*^	91.14 ^*de*^	8.17 ^*e*^	58.68 ^*de*^
100	260.86 ^*bc*^	349.07 ^*b*^	188.78 ^*cd*^	460.48 ^*a*^
Mean	166.24 ^*A*^	150.96 ^*A*^	66.04 ^*B*^	182.65 ^*A*^

Values that did not differ significantly in the SNK test (p < 0.001) are marked with the same letters in columns

### Yeast survival under exposure to propiconazole

Fifteen out of the 16 tested isolates developed only at the lowest concentration of propiconazole (100 μl dm^− 3^) (Table 2). Isolate *Rh. glutinis* 59 survived under exposure to a propiconazole dose of 200 μl dm^− 3^. None of the analyzed isolates survived under exposure to propiconazole doses of 300, 400 and 500 μl dm^− 3^.

### Propiconazole degradation by yeasts

All of the tested yeast isolates reduced the content of propiconazole in a water solution (Table 4). The greatest reduction in propiconazole content was induced by isolates *Rh. glutinis* Rg 55 (74.5%) and *Rh. glutinis* Rg 92 (38.5%), relative to the initial content of propiconazole. Isolates of *A. pullulans* decreased propiconazole content by 8.6, 12.1 and 12.2%, whereas isolates of the genus *Cryptococcus* - by 9.8 and 23.3%.

**Table 4:** Propiconazole degradation by yeast isolates

Isolates	Reduction in propiconazole content in %
*A. pullulans* 203	12.06 ^*f*^
*A. pullulans* 83	12.15 ^*f*^
*A. pullulans* 137	8.58 ^*d*^
*Cryptococcus* sp. 48	9.84 ^*e*^
*Cryptococcus* sp. C123	23.25 ^*c*^
*Rh. glutinis* Rg 55	74.45 ^*a*^
*Rh. glutinis* Rg 92	38.48 ^*b*^

Values that did not differ significantly in the SNK test (p < 0.001) are marked

With the same letters in columns

Ability of yeast isolates to minimize the phytotoxic effects of the Bumper 250EC fungicide on wheat seedlings.

**Fig. 4:**
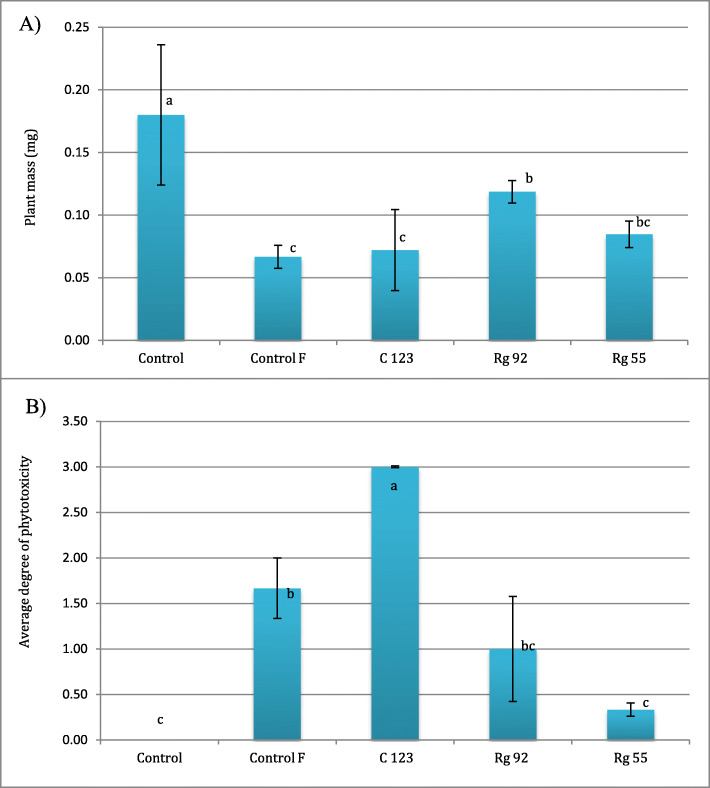
Dry matter of wheat seedlings (**a**) and phytotoxic effects of of propiconazole (**b**) on seeds dressed with yeasts. Control – control without fungicide, Control F – control with fungicide; C 123 – seeds dressed with *Cryptococcus* sp. C 123 isolate; Rg 92 – seeds dressed with *Rhodotorula glutinis*; Rg 92 and Rg 55 isolates; Rg 55 - seeds dressed with *Rhodotorula glutinis* Rg 55 isolate

The dry matter content of wheat seedlings grown in soil contaminated with the Bumper 250EC fungicide was reduced 2.7-fold, their leaves were yellow and smaller than the leaves of control plants (Fig. 4). Isolates *Rh. glutinis* Rg 92 and *Rh. glutinis* Rg 55 decreased the fungicide’s phytotoxic effects. The first isolate contributed to a significant increase in the dry matter content of seedlings (1.8-fold relative to Control F with fungicide), whereas the second isolate significantly reduced the severity of chlorosis (5-fold relative to Control F with fungicide).

## Discussion

Leaf surfaces do not offer a supportive environment for the development of yeasts. In this study the counts of yeasts in control ranged from 0.1 to 4.1 log (CFU + 1) and were determined mainly by the wheat growth stage. Generally the yeast epiphyte counts noted in this study were lower than those reported by in wheat by Fokkema et al. [6, 42] and Dik et al. [7], but similar to those noted on the leaves of *Oxalis acetosella* [43]*. Similarly to our study, I*nacio et al. [44] and Glashakova and Chernov [43] reported a predominance of *Rhodotorula* spp., and Fokkema and Van Der Meulen [43] observed a predominance of *Aureobasidium pullulans* in the wheat phyllosphere. Yeasts that enter into positive interactions with crop plants and protect them against pathogens have many potential applications in biological crop protection, and their abundance can influence plant health [45, 46, 47, 48, 49].

In the current field experiment, the tested triazole fungicides (propiconazole, epoxiconazole) and commercial mixtures of triazoles and benzimidazoles (flusilazole and carbendazim) or strobilurin (fluoxastrobin and prothioconazole) generally did not decrease the density of yeast communities or their inhibitory effects were short-lived. Yeast communities were rebuilt and their density increased, which can probably be attributed to an increase in the abundance of resistant species and strains. Dickinson and Wallace [3] observed a significant reduction in the population size of pink yeasts and all yeasts on cereal leaves sprayed with benzimidazole fungicides. Long-term benzimidazole use in cereals probably contributed to the emergence of resistant strains, and an abundance of yeast communities resistant to carbendazim was reported on wheat leaves by Fokkema et al. already in 1987 [6]. Buck and Burpee [50] also found that yeast isolates that had not been previously exposed to fungicides were more sensitive to chlorothalonil, propiconazole, flutolanil and iprodione than yeasts isolated from protected lawn grass. According to the cited authors, yeast communities in the phylloplane of grasses are most often resistant to fungicides. Fungicide-resistant mutants are present in fungal populations already before exposure to these xenobiotics [51, 52]. Yeasts’ resistance to dithiocarbamate fungicides can result from the overexpression of heat shock genes or the expression of genes encoding multidrug resistance proteins, such as Pdr5p and Flr1p [49, 53]. Mutations in the cytochrome b (*CYTB*) gene, the target site of strobilurin fungicides, are also often noted [54]. Protective mechanisms against azole fungicides in yeasts include the activation of membrane transporters, changes in the cell membrane structure or mutations in the *CYP 51* gene which encodes lanosterol 14α-demethylase, the key enzyme inhibited by azoles [55].

In this study, nearly 44% of *Rh. glutinis* isolates were insensitive to all tested fungicides in the disk diffusion test. Complete fungicide resistance was noted in 16% of *A. pullulans* isolates. Our findings are consistent with the findings of Karlsson et al. [9] who observed that the use of fungicides was significantly correlated with changes in the relative abundance of selected fungal taxa on wheat leaves. In a study evaluating the sensitivity of yeasts colonizing grapes, Comitini and Ciani [2] reported a predominance of *Hanseniaspora uvarum* on unprotected fruit. Grapes protected with fungicides were colonized predominantly by *Aureobasidium pullulans* and *Cryptococcus* spp. Cadez et al. [1] demonstrated that several yeast species isolated from grapes were characterized by varied sensitivity to iprodione, pyrimethanil and fludioxonil in vitro. In a study by Karlsson et al. [9], fungicide treatments decreased the biological diversity of fungal communities on wheat leaves. In agoecosystems, fungal taxa can also be closely interconnected by unknown functional interactions, leading to pairwise co-occurrence [9]. Fungicides can also modify plant physiology and the availability of nutrients [56].

Yeast isolates differed in sensitivity to the analyzed fungicides in the disk diffusion test. Flusilazole, carbendazim (Alert 375SC) and epoxiconazole (Soprano125SC) were the most toxic compounds. Propiconazole (Bumper 250EC) was characterized by lower toxicity, whereas the commercial mixture of fluoxastrobin and propiconazole (Fandango 200 EC) was least toxic for yeasts. Similar observations were made by Lima et al. [46] in whose study, *Rh. glutinis* isolates were resistant to strobilurin fungicides, but sensitive to azoles. Nagy et al. [57] demonstrated that *Rh. slooffiae* was sensitive to selected fungicides, including azoles. Lima et al. [46] and Vero et al. [58] reported that *A. pullulans* was highly resistant to strobilurins, benzimidazoles and selected azoles.

In the present study, the *Rh. glutinis* Rg 55 isolate was exceptionally effective in degrading propiconazole whose content in an aqueous solution decreased by 74.4% after 48 h. *Rhodotorula glutinis* oxidized phenanthrene in a study by Gupta et al. [59] and degraded patulin in the experiment conducted by Castoria et al. [60]. Romero et al. [61] identified *Rh. glutinis* isolates degrading biphenyls. The propiconazole-degrading ability of *Rh. glutinis* has been demonstrated for the first time in this study. To date, only bacterial isolates capable of degrading this fungicide have been described in the literature. Sarkar et al. [62] demonstrated that propiconazole can be eliminated in up to 72.8% by *Pseudomonas putida* bacteria (22.7–72.8%, subject to bacterial strain). Satapute and Kaliwal [34] observed that propiconazole can also be degraded by *Pseudomonas aeruginosa* bacteria with an active *CYP450* gene encoding various enzymes.

In the presented greenhouse experiment, wheat seeds sown in soil contaminated with Bumper 250EC germinated at a significantly lower rate, and wheat seedlings were characterized by lower dry matter content. The above observations confirm that the tested fungicide exerted phytotoxic effects. Petit et al. [56] concluded that azoles can exert both growth-stimulating and phytotoxic effects on plants. According to many authors, triazoles can positively influence plants by boosting their resistance to pathogens through enhanced synthesis of phytoalexins, cell wall lignification and stimulation of enzymes that synthesize phenolic compounds [63, 64]. Wu and Von Tiedemann [65] observed that azoles can increase plant resistance to salinity by stimulating the synthesis of antioxidant enzymes. In their study, epoxiconazole delayed ageing in *Triticum aestivum* by increasing the plant’s antioxidant potential. The environmental toxicity of propiconazole can be attributed mainly to its persistence in soil [66]. Intensive fungicide use contributes to the accumulation of fungicides in the environment and food products [67]. In the present greenhouse experiment, the symptoms of chlorosis in wheat seedlings grown in contaminated soil were reduced by all tested isolates, and the endophytic isolate *Rh. glutinis* Rg 55 was most effective. Similar observations were made by Rai et al. [68] who found that endophytic fungi promoted plant growth and resistance to biotic and abiotic stresses by producing plant-stimulating hormones and bioactive metabolites.

## Conclusions

Yeast communities colonizing wheat leaves are eliminated selectively by the applied fungicides due to differences in the sensitivity of yeast species or strains within a species. The group of fungicide-resistant yeast isolates includes strains capable of degrading propiconazole. These strains can potentially protect plants against the phytotoxic effects of fungicides and accelerate fungicide degradation in agroecosystems, and enhance the growth of seedlings treated with chemical protection agents.

## Methods

### Field-plot experiment

A three-year field-plot experiment was conducted in 2009–2011 in Tomaszkowo (N: 53° 42′, E: 20° 22′) in north-eastern Poland. Plots were sown with winter wheat cv. Bogatka (Danko Plant Breeding in Chorynia, Poland, https://www.danko.pl/odmiany/bogatka). Two crop protection strategies for protecting crops against *Zymoseptoria tritici,* the most dangerous pathogen of wheat leaves in Europe, were analyzed (Table 5). Fungicides Bumper 250 EC (Fung 1, propiconazole) and Fandango 200 EC (Fung 3, commercial mixture of fluoxastrobin and propiconazole) were applied in first node at least 1 cm above tillering (BBCH 31) [69] and fungicides Alert 375SC (Fung 2, commercial mixture of flusilazole and carbendazim) and Soprano 125SC (Fung 4, epoxiconazole) were applied in the heading stage (BBCH 55) in the doses specified in Table 5. Plot size was 20 m^2^, and the experiment had a random block design with four replications. Plots sprayed with 400 l/ha of water were the control.

**Table 5:** Fungicide treatments in winter wheat

Treatment Control	Trade name (dose)	Application date
	Water	Water
Fungicide 1 (Fung 1)	Bumper 250 EC ^1^ (0.5 l/ha)	First node at least 1 cm above tillering node (BBCH 31)
Fungicide 2 (Fung 2)	Alert 375 SC ^2^ (1 l/ha)	First node at least 1 cm above tillering node (BBCH 31)
Fungicide 3 (Fung 3)	Fandango 200 EC ^3^ (1 l/ha)	Middle of heading (BBCH 55)
Fungicide 4 (Fung 4)	Soprano 125 SC ^4^ (1 l/ha)	Middle of heading (BBCH 55)

^1^ – propiconazole - 25.1% (Makhteshim Chemical Works Ltd., Israel), ^2^ – flusilazole – 125 g l^−1^, carbendazim – 250 g l^− 1^ (Du Pont International Operations Sarl, Switzerland), ^3^ – fluoxastrobin - 100 g l^− 1^, prothioconazole – 100 g l^− 1^ (Bayer SAS, France), ^4^ – epoxiconazole – 125 g l^− 1^ (Makhteshim Chemical Works Ltd., Israel)

Wheat leaves were sampled six times during the growing season, including three times after every fungicide treatment: after 24 h, 10 days and 20 days. The third fully developed topmost leaf was sampled from each plant. Leaf segments with a length of 1 cm were cut out at a distance of 2 cm from the leaf base. The width of leaf segments was measured.

### Yeast isolation and identification

Yeasts were isolated immediately after leaf sampling. Microorganisms were isolated from 15 1-cm-long leaf segments that were surface disinfected by immersion in 1% sodium hypochlorite for 1 min and macerated in a mortar with 1 cm^3^ of sterile water. The second microbial dilution of the suspension in the amount of 0.1 cm^3^ was collected from the mortar and was pour-plated on Martin agar [70]. The experiment was performed in three replications. Yeast were incubated for 7 days in darkness at a temperature of 24 °C. The colonies were counted and expressed per 1 cm^2^ of leaf area based on leaf width and 10^− 2^ dilution. Randomly selected yeast colonies with a varied morphology were isolated from plates. They were pour plated on potato dextrose agar (PDA, Merck, Poland) with the addition of kanamycin (A&A Biotechnology, Poland) and streptomycin (Sigma, Poland). For short-term storage at a temperature of 4 °C, yeast isolates and the substrate were placed in 1.5 cm^3^ Eppendorf tubes protected with sterile oil. For long-term storage at − 80 °C, yeast isolates were placed in cryogenic vials (Biomaxima, Poland).

Yeast isolates were identified based on morphological features, the size of budding cells, pseudofilaments and chlamydospores under a microscope at 400 x magnification (Nikon 200 E, Japan) according to the available keys and monographs [71, 72]. A total of 75 yeast isolates were isolated for further analyses (Table 2).

Yeast DNA was isolated with the DNA Genomic Mini AX YEAST Kit (A&A Biotechnology, Poland). The fragment with ITS 1, 5.8S and ITS 2 rDNA regions was amplified with specific ITS5 (F) GTATCGGACGGAGATCCAGC and ITS4 (R) TTGCTCAGTGCATTGTCGG primers [73] with the FailSafe PCR Kit (Epicentre, Poland). The PCR reaction was performed on 20 ng of DNA in the Mastercycler Ep Gradient thermal cycler (Eppendorf, USA). The reaction had the following thermal profile: 3 min at 95 °C, followed by 34 cycles of: 1 min at 95 °C, 1 min at 58 °C, 3 min at 74 °C and 10 min at 74 °C. PCR amplicons with 0.5 μg/ml of ethidium bromide were subjected to electrophoresis in 1% agarose gel (Prona NU Micropor, Poland) in TBE buffer (Blirt S.A., Poland). Electrophoresis separation products were visualized with a transilluminator (MultiDoc-It, USA). Amplicons were sequenced by the Institute of Biophysics and Biochemistry of the Polish Academy of Sciences in Warsaw (www.ibb.waw.pl). The similarities between sequences were determined with NCBI BLAST (National Center for Biotechnology Information, Basic Local Alignment Search Tool, http://blast.ncbi.nlm.nih.gov/Blast.cgi).

### Fungicide toxicity for yeast isolates in the disk diffusion test

Seventy-five yeast isolates were spread plated on PDA in Petri plates, including 47 isolates of *A. pullulans*, 18 isolates of *Rh. glutinis*, 6 isolates of *Cryptococcus* sp., 2 isolates of *D. hansenii* and 1 isolate of *C. albicans* and *Pseudozyma* sp. each. Paper discs (Biomaxima, Poland) with a diameter of 5 mm were saturated with propiconazole, epoxiconazole, the flusilazole and carbendazim mixture and the fluoxastrobin and prothioconazole mixture, and were plated [74]. The tested fungicides were applied at 1, 10, 100 μl dm^− 3^ water based on the concentration of the active ingredient. The size of the inhibition zone was measured after 4 days in the ImageJ 1.48p program [75]. The results were presented as the area of the inhibition zone in square millimeters. The isolates were divided into three groups based on their sensitivity to fungicides. Non-sensitive (NS) isolates did not produce inhibition zones around paper discs saturated with fungicides. The inhibition zones formed by sensitive (S) isolates had an area of 1 to 250 mm^2^, whereas highly sensitive (HS) isolates produced inhibition zones with an area larger than 250 mm^2^.

### Yeasts ability to biodegrade propiconazole

Yeast isolates that were not sensitive to propiconazole in the disk diffusion test were exposed to this fungicide in a liquid medium. Suspensions of 16 yeast isolates (1 cm^3^ each) with cell density of 10^8^ were placed in 15 cm^3^ flasks (Bionovo, Poland) containing 9 cm^3^ of a liquid medium (beef extract – 1 g, soy peptone – 5 g, NaCl – 5 g, glucose – 1 g, yeast extract – 7 g dm^− 3^ water). Propiconazole was added to the flasks in the following amounts: 100, 200, 300, 400 and 500 μl dm^− 3^. After 48 h of incubation at 24 °C, 100 μl of every yeast suspension was pour plated on PDA. Flasks without propiconazole were the control. The experiment was performed in three replications.

Seven yeast isolates obtained from wheat leaves, non-sensitive to fungicides, were selected for the propiconazole biodegradation test: *Rh. glutinis* Rg 55, *Rh. glutinis* Rg 92, *A. pullulans* 203, *Cryptococcus* sp. 48, *Cryptococcus* sp. C 123, *A. pullulans* 83 and *A. pullulans* 137 (Table 2). The tested isolates were cultured in 10 cm^3^ of water with the addition of 2 mg dm^3^ of propiconazole (Sigma, Poland) for 48 h at a temperature of 27 °C. The microorganisms were centrifuged (8000 rpm, 5 min), and propiconazole residues were extracted and analyzed by HPLC [62] in the Agilent Technologies 1200 Series HPLC System (USA) with a diode array detector. The analysis was performed in isocratic elution mode with a mixture of acetonitrile and water (80/20, v/v) acidified with 0.15% formic acid as the eluent, at a flow rate of 0.5 cm^3^ /min. Chromatographic separation was performed on the Agilent Eclipse XDB-C18 column (150 mm × 4.6 mm, 5 μm) at 30 °C. Chromatographic data were registered at 220 nm wavelength for 11 min. Data were acquired and analyzed based on the calibration curve for the propiconazole standard in the HP ChemStation program. The experiment was conducted in two replications.

### Application of yeasts to decrease propiconazole’s toxic effects on wheat seedlings

The seeds of winter wheat cv. Bogatka dressed with three yeast isolates were sown in pots with a diameter of 12 cm, filled with 160 g of sterile soil (autoclaved twice at 121 °C and 1.2 atm), in a greenhouse. Seeds were dressed with *Cryptococcus* sp. C 123, *Rh. glutinis* Rg 55 and *Rh. glutinis* Rg 92 isolates which most effectively degrade propiconazole. Seeds were dressed by immersion in a yeast suspension of 10^6^ cells cm^− 3^ water for 30 min. Germinated seeds were watered every 48 h with the Hoagland solution containing macronutrients and micronutrients [76], in the amount of 30 cm^3^ per pot, throughout the experiment. After 14 days, seedlings were treated with the Bumper 250EC fungicide at a concentration 50-fold higher than that recommended by the manufacturer (6.25% solution in 20 cm^3^ of water per pot). Plants watered only with the Hoagland solution (Control) and fungicide-treated plants growing from seeds that were not treated with the yeast suspension (Control F) were the control. The experiment was conducted in four replications. The fungicide’s phytotoxic effects were evaluated after two weeks. Symptoms of leaf chlorosis were evaluated on a 5-point scale: 0 points – no chlorosis, 1 point – chlorosis affecting 10% of leaf area, 2 points – chlorosis affecting 10–30% of leaf area, 3 – chlorosis affecting 30–50% of leaf area, 4 points – chlorosis affecting 50–90% of leaf area, 5 points – chlorosis affecting more than 90% of leaf area. The dry matter content of seedlings was determined.

### Statistical analysis

The results were processed using the Statistica 13.0 (2016) software package (Statistica 13.0 [77] software). Yeast colony counts were log transformed (CFU + 1), and the results were presented as log (CFU + 1) per 1 cm^2^ of leaf. The data were subjected to analysis of variance (ANOVA), and the significance of differences between mean values was determined by the Student-Newman-Keuls (SNK) test. The size of inhibition zones in the diffusion test was expressed in mm^2^. The significance of differences between the mean areas of inhibition zones was determined by the Student-Newman-Keuls (SNK) test. The percentage of isolates of yeast species with varied sensitivity to the tested agrochemicals was presented as heat maps for each product [78].

## Data Availability

The dataset used and analyzed during the current study are available from corresponding author on reasonable request.

## Abbreviations

ANOVA: Analysis Of Variance
BBCH: Biologische Bundesanstalt, Bundessortenamt and Chemical Industry (German scale used to identify the phenological development stages of a plant
BLAST: Basic Local Alignment Search Tool
CFU: Colony Forming Unit
DNA: DeoxyriboNucleic Acid
EDTA: EthyleneDiamineTetraacetic Acid
ITS: Internal Transcribed Spacer
NCBI: National Center for Biotechnology Information
PCR: Polymerase Chain Reaction
PDA: Potato Dextrose Agar
TBE: Tris Borate EDTA
SNK: Student-Newman-Keuls
